# Emicizumab prophylaxis in infants with hemophilia A (HAVEN 7): primary analysis of a phase 3b open-label trial

**DOI:** 10.1182/blood.2023021832

**Published:** 2023-12-23

**Authors:** Steven W. Pipe, Peter Collins, Christophe Dhalluin, Gili Kenet, Christophe Schmitt, Muriel Buri, Víctor Jiménez-Yuste, Flora Peyvandi, Guy Young, Johannes Oldenburg, Maria Elisa Mancuso, Kaan Kavakli, Anna Kiialainen, Sonia Deb, Markus Niggli, Tiffany Chang, Michaela Lehle, Karin Fijnvandraat

**Affiliations:** 1 University of Michigan, Ann Arbor, MI; 2 School of Medicine, Cardiff University, Cardiff, United Kingdom; 3 F. Hoffmann-La Roche Ltd, Basel, Switzerland; 4 Sheba Medical Center, Ramat Gan, Israel; 5 Tel Aviv University, Tel Aviv, Israel; 6 La Paz University Hospital-IdiPaz, Autónoma University, Madrid, Spain; 7 Fondazione IRCCS Ca’ Granda Ospedale Maggiore Policlinico, Angelo Bianchi Bonomi Hemophilia and Thrombosis Center, Milan, Italy; 8 Università degli Studi di Milano, Milan, Italy; 9 Children’s Hospital Los Angeles, Los Angeles, CA; 10 Institute of Experimental Hematology and Transfusion Medicine, University Hospital Bonn, Medical Faculty, University of Bonn, Bonn, Germany; 11 IRCCS Humanitas Research Hospital, Rozzano, Milan, Italy; 12 Humanitas University, Pieve Emanuele, Milan, Italy; 13 Ege University Children’s Hospital Department of Hematology, Bornova, İzmir, Turkey; 14 Genentech, Inc, South San Francisco, CA; 15 Spark Therapeutics, Inc, San Francisco, CA; 16 University of Amsterdam, Amsterdam, The Netherlands

## Abstract

Subcutaneous emicizumab enables prophylaxis for people with hemophilia A (HA) from birth, potentially reducing risk of bleeding and intracranial hemorrhage (ICH). HAVEN 7 (NCT04431726) is the first clinical trial of emicizumab dedicated to infants, designed to investigate the efficacy, safety, pharmacokinetics, and pharmacodynamics of emicizumab in those aged ≤12 months with severe HA without factor VIII (FVIII) inhibitors. Participants in this phase 3b trial received emicizumab 3 mg/kg maintenance dose every 2 weeks for 52 weeks and are continuing emicizumab during the 7-year long-term follow-up. Efficacy end points included annualized bleed rate (ABR): treated, all, treated spontaneous, and treated joint bleeds. Safety end points included adverse events (AEs), thromboembolic events (TEs), thrombotic microangiopathies (TMAs), and immunogenicity (anti-emicizumab antibodies [ADAs] and FVIII inhibitors). At primary analysis, 55 male participants had received emicizumab (median treatment duration: 100.3; range, 52-118 weeks). Median age at informed consent was 4.0 months (range, 9 days to 11 months 30 days). Model-based ABR for treated bleeds was 0.4 (95% confidence interval, 0.30–0.63), with 54.5% of participants (n = 30) having zero treated bleeds. No ICH occurred. All 42 treated bleeds in 25 participants (45.5%) were traumatic. Nine participants (16.4%) had ≥1 emicizumab-related AE (all grade 1 injection-site reactions). No AE led to treatment changes. No deaths, TEs, or TMAs occurred. No participant tested positive for ADAs. Two participants were confirmed positive for FVIII inhibitors. This primary analysis of HAVEN 7 indicates that emicizumab is efficacious and well tolerated in infants with severe HA without FVIII inhibitors.

## Key Points

• Subcutaneous emicizumab administered from birth has the potential to reduce the risk of ICH and joint bleeds before damage occurs.
• Primary analysis of the HAVEN 7 trial indicates emicizumab is efficacious and well tolerated in infants with severe HA.

## Visual Abstract

**Figure:**
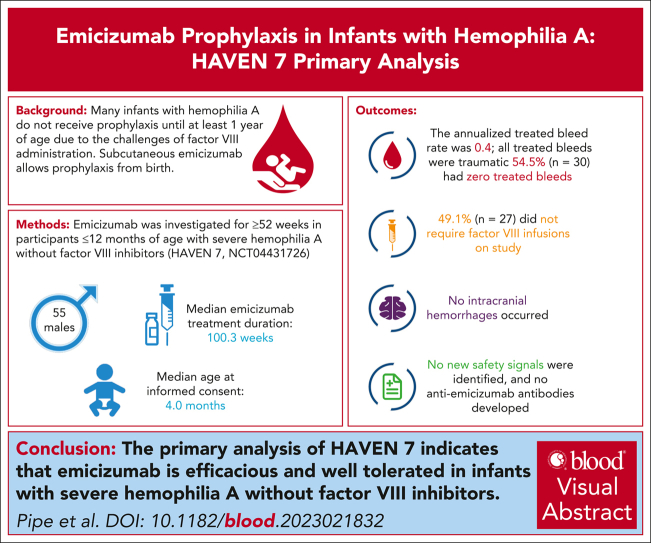

## Teaser

Intravenous prophylaxis with factor VIII (FVIII) for infants under 1 year of age with severe hemophilia can be logistically challenging. Emicizumab is given subcutaneously and acts as an immunoglobulin G molecular bridge between activated FIX and FX, thereby substituting for the important role of FVIII. Pipe and colleagues report on the efficacy and safety of emicizumab in 55 infants in a single-arm study, finding high compliance and low bleeding rates with no spontaneous bleeds and no immunogenicity. These data indicate the potential of emicizumab prophylaxis initiated very early in life to reduce the morbidity of severe hemophilia.

## Introduction

Congenital hemophilia A (HA) is an X-linked hereditary disorder characterized by a deficiency of factor VIII (FVIII), which increases the risk of frequent bleeding into joints, muscles, and soft tissues, often without evident trauma or injury.^1^ Severity of HA can be classified by endogenous FVIII levels, although this categorization does not uniformly correlate with bleeding phenotype.^2^ Severe HA (intrinsic FVIII levels <1%) is usually associated with high bleeding frequency from early childhood.^3^

In children, and especially infants, every bleed matters, because the damage incurred accrues early in life and may lead to joint arthropathy and disability in adulthood.^4^ Furthermore, infants with HA not receiving prophylaxis have a 33-times higher risk of life-threatening intracranial hemorrhages (ICHs) than infants without hemophilia, with the potential of developing severe long-term sequelae.^5^^,^^6^ For this reason, starting prophylaxis very early in life should be the standard of care.^1^ Until recently, prophylaxis has required intravenous FVIII replacement.^7^ However, the frequent (2-4 times weekly) infusions mean many young children have a central venous access device (CVAD) inserted to facilitate frequent, regular, and long-term venous access and reduce the treatment burden.^8^^,^^9^ Because the use of CVADs is associated with complications such as infections or thrombosis,^9^ prophylaxis is often delayed until after the first year of life to access peripheral veins without the need for the insertion of a device.^10^ However, there is substantial risk of ICH in the first year of life (2.1% incidence per 100 live births of infants with hemophilia in the neonatal period alone; 95% confidence interval [CI], 1.5-2.8),^5^^,^^11^ and joint damage can also occur during this untreated period.^4^ Moreover, ∼30% of people with severe HA receiving FVIII develop inhibitors, at a median age of 15.5 months after a median of 9 to 36 exposure days (EDs) to FVIII treatment.^12^^,^^13^ This reduces the benefits of FVIII therapy and worsens their clinical outcomes.^14^^,^^15^

Subcutaneous administration of emicizumab allows for prophylaxis initiation at a very young age, with a potential to reduce bleeds and life-threatening ICH while avoiding complications associated with CVADs. Emicizumab is a recombinant, humanized, bispecific monoclonal antibody that bridges activated FIX and FX to substitute for the function of deficient activated FVIII.^16^^,^^17^ Based on the results of the phase 3 HAVEN clinical trial program, in which >500 participants have been enrolled and treated to date,^18^, ^19^, ^20^, ^21^, ^22^, ^23^ emicizumab is indicated as routine prophylaxis for people of all ages with HA in many regions, including the United States and Japan.^16^ In the European Union, emicizumab is indicated as routine prophylaxis for people of all ages with FVIII inhibitors and those without FVIII inhibitors if they have severe HA or moderate HA with severe bleeding phenotype.^17^ At all approved dose regimens, emicizumab offers stable and sustained therapeutic plasma concentrations.^24^

Limited experience with early emicizumab prophylaxis has been reported in clinical trials and real-world data. Two reported trials of emicizumab have included children: HAVEN 2 (85 children aged 14 months to 11 years) and HOHOEMI (13 children aged 4 months to 10 years).^19^^,^^25^ These trials included 11 children aged <2 years, including 1 child aged <1 year.^19^^,^^25^ Real-world data on emicizumab use for infants are mostly case series and reports,^26^, ^27^, ^28^ yet they are consistent with the efficacy and safety profile observed in older children with HA in HAVEN 2 and HOHOEMI.^19^^,^^25^ A population pharmacokinetic (PK) model, performed to characterize the PK of emicizumab in people with HA enrolled in phase 1 to 3 trials, predicted lower exposure that remains at the plateau of the exposure-response relationship in newborns.^24^^,^^29^^,^^30^ Based on this modeling, the approved dosing of emicizumab applies for people with hemophilia A of all ages, including infants.

Building on these early experiences, the aim of the HAVEN 7 trial is to investigate the efficacy, safety, PK, and pharmacodynamics (PD) of emicizumab in infants from birth to age ≤12 months. Here, we report the primary analysis results.

## Methods

### Study design and participants

HAVEN 7 (NCT04431726) is a phase 3b, multicenter, open-label, single-arm trial of emicizumab in infants with severe congenital HA (intrinsic FVIII level <1%) without FVIII inhibitors (<0.6 Bethesda units [BU]/mL and no documented history). Eligible participants included newborns to infants aged ≤12 months weighing ≥3 kg at the time of informed consent. Participants were previously untreated (PUPs) or minimally treated (MTPs). MTPs were defined as having 1 to 5 EDs (defined as a calendar day when ≥1 dose was received by an individual) with hemophilia-related treatment containing FVIII, such as plasma-derived FVIII, recombinant FVIII, fresh/frozen plasma, cryoprecipitate, or whole blood products. Those who had only received antifibrinolytics were still considered to be PUPs. Participants had no evidence of active ICH and had normal hematologic, hepatic, and renal function (definitions are provided in the supplemental Data, which is available on the *Blood* website). Full inclusion/exclusion criteria are provided in the supplemental Data.

The objective of HAVEN 7 was to evaluate the efficacy, safety, PK, and PD of emicizumab prophylaxis in this population, administered subcutaneously at 3 mg/kg once weekly for 4 weeks as a loading dose, followed by maintenance dosing of 3 mg/kg once every 2 weeks for a total of 52 weeks. The protocol was approved by the institutional review board/ethics committee at each site, and the trial was conducted in accordance with the International Conference on Harmonisation Guidelines for Good Clinical Practice, the Declaration of Helsinki, and all applicable regulations.

After 52 weeks of treatment, participants continued to receive emicizumab 3 mg/kg once every 2 weeks or could switch to 1.5 mg/kg once weekly or 6 mg/kg once every 4 weeks for the 7-year follow-up period. Participants with >2 qualifying bleeds within a 12-week interval could have their dose up-titrated to 3 mg/kg once weekly from week 17. Qualifying bleeds were defined as spontaneous, clinically significant, clinician verified (eg, with diagnostic imaging, clinical examination, or a photograph), and occurring while receiving emicizumab maintenance.

### Objectives

The efficacy objective of HAVEN 7 was to evaluate emicizumab based on bleed end points, including treated bleed rate, all bleed rate, treated spontaneous bleed rate, and treated joint bleed rate (definitions in the supplemental Data). For participants whose dose was up-titrated, only efficacy data before up-titration are included. Joint health will be assessed during the long-term follow-up period in participants aged ≥4 years; the Hemophilia Joint Health Score 2.1 and additive magnetic resonance imaging scale score of the International Prophylaxis Study Group of specific joints will aid in evaluating preservation of joint health in the setting of earliest initiation of prophylaxis.^31^^,^^32^ Participants’ Hemophilia Joint Health Score 2.1 will be measured annually from year 4 to 8, and a bilateral magnetic resonance imaging of the knees, ankles, and elbows will be performed at years 5 and 8.

Safety assessments included incidence of adverse events (AEs; and severity according to the World Health Organization toxicity grading scale), thromboembolic events or thrombotic microangiopathies, AEs leading to emicizumab discontinuation, injection-site reactions, severe hypersensitivity, anaphylaxis, and anaphylactoid events.

Emicizumab PK profile was characterized based on plasma trough concentrations of emicizumab. The biomarker end points included PD parameters (activated partial thromboplastin time [aPTT], thrombin generation [TG] peak height, and FVIII-like activity), and concentrations of emicizumab targets (antigen levels of FIX and FX).

Immunogenicity end points comprised the incidence and significance of anti-emicizumab antibodies (ADAs) and de novo development of FVIII inhibitors.

### Assessments and data collection

Medical history, including clinically significant diseases, procedures, allergies, history of anaphylaxis, or known thrombophilia since birth, were recorded on the electronic case report form (eCRF). All bleeds experienced since birth and before enrolling in the trial on day 1 were documented on the eCRF.

Medication administered to the participant from 4 weeks before enrollment through study completion or safety follow-up was reported on the concomitant medications eCRF. Hemophilia-related treatments administered to participants since birth and before enrolling in the trial were documented on the eCRF.

On-study bleed and medication-related observer-reported outcome data were collected at home or in the clinic throughout the trial. Emicizumab administration, bleeds experienced, and hemophilia-related treatments received by a participant were reported by parents/caregivers through a bleed and medication questionnaire on an electronic handheld device provided at enrollment. Parents/caregivers were asked to record and confirm these data at least weekly, and data entered since a participant’s previous clinic visit were reviewed for completeness and accuracy by the parents/caregivers and the investigator at subsequent visits.

PK and biomarkers were assessed once every 2 weeks during weeks 1 to 9 and once every 4 weeks during weeks 13 to 53 before emicizumab administration. Trough plasma concentrations of emicizumab were measured by validated enzyme-linked immunosorbent assay.^24^ aPTT, TG, FVIII activity (using a chromogenic assay containing human FIX and FX, considered FVIII-like activity), and FIX and FX antigen concentrations were assessed as previously described.^33^ FVIII-like activity does not represent a true FVIII equivalence due to different biochemical properties of the proteins but can be used to measure the relative PD effect of emicizumab.

ADAs were measured by bridging enzyme-linked immunosorbent assay in all participants at baseline^34^; on study at weeks 1, 5, 17, 29, 41, and 53; and during long-term follow-up in case of clinical suspicion. FVIII inhibitors were measured by chromogenic Bethesda assay in MTPs at baseline.^35^ FVIII inhibitor development was tested for after any 3 FVIII EDs or a block of EDs (defined as a minimum of 2 consecutive FVIII doses).

Parents/caregivers could withdraw the participant voluntarily from the trial at any time for any reason, and investigators could withdraw a participant for reasons listed in the supplemental Data.

### Statistical analysis

No formal hypothesis testing was planned; all analyses were descriptive. No adjustment for multiplicity of end points was considered.

The sample size was based on recruitment feasibility and clinical, rather than on statistical, considerations in view of the limited number of infants from birth to aged ≤12 months available for participation in clinical trials, and to collect sufficient data to assess the efficacy, safety, PK, and PD of emicizumab in this population.

Bleed data are presented as calculated median (interquartile range) annualized bleed rates (ABRs) using individual ABRs^23^ and model-based ABRs using a negative binomial regression model, which takes into account variations in participant follow-up time.^36^

The primary analysis was planned to be performed when the last participant had completed 52 weeks on the study, was lost to follow-up, or had withdrawn from trial treatment, whichever came first.

## Results

### Participant characteristics

**Figure 1.**
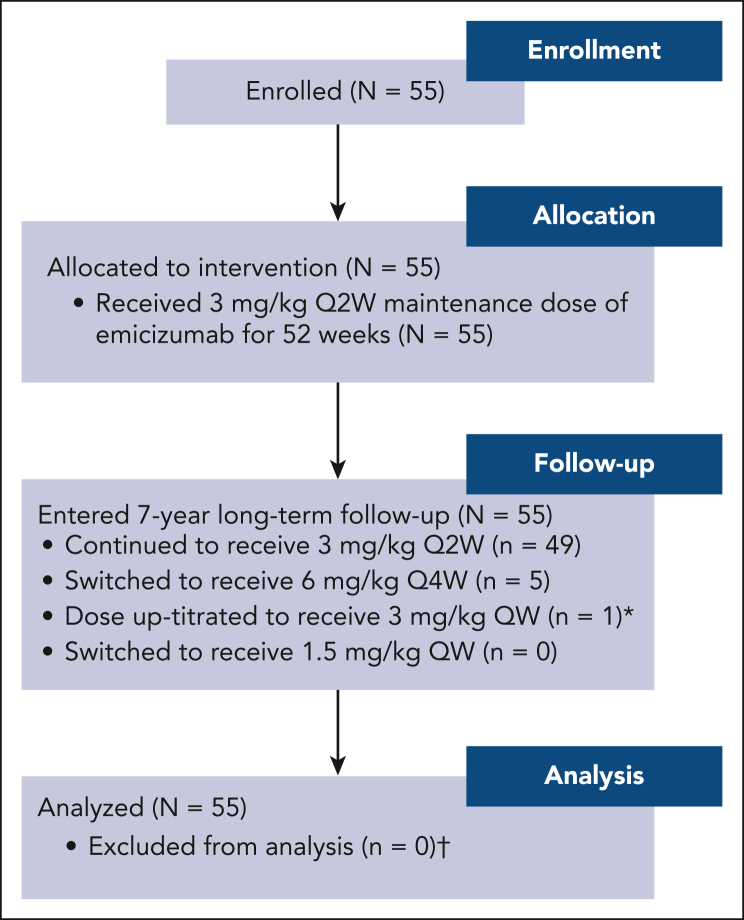
**Participant disposition.** ∗ indicates that emicizumab dose was up-titrated in 1 participant on day 374 while starting the long-term follow-up period. Up-titration was per investigator request based on locally assessed decreasing emicizumab levels (confirmed retrospectively to be 6.6 μg/mL in a central assessment). This participant experienced 3 treated and 2 untreated bleeds (all traumatic) before and after up-titration, respectively. † indicates that bleed end points consider data before up-titration only. Q2W, every 2 weeks; Q4W, every 4 weeks; QW, weekly.

In total, 55 male participants were recruited between February 2021 and May 2022 (Figure 1). All participants completed 52 weeks in the study by receiving emicizumab prophylaxis 3 mg/kg once every 2 weeks and have entered the long-term follow-up period. Upon entering follow-up, 49 participants (89.1%) continued to receive emicizumab 3 mg/kg once every 2 weeks, 5 (9.1%) switched to 6 mg/kg once every 4 weeks, and 1 was up-titrated to 3 mg/kg once weekly.

At the clinical cutoff date (CCOD) for this analysis (22 May 2023), median treatment duration (date of the last dose of study medication minus the date of the first dose, plus 1 day) before up-titration was 100.3 (range, 52-118) weeks. Overall, participant compliance with the expected emicizumab dosing was high, with only 1 participant missing doses (no safety events or bleeding were reported during this period) and 2 participants receiving less than the full dose at 1 time point each. The median age at informed consent was 4.0 months (range: 9 days to 11 months 30 days; Table 1); median age at CCOD was 29 (range, 12-39) months. Approximately half (n = 25 [45%]) of the participants were aged 0 to <3 months at time of informed consent; 30 (54.5%) were aged 3 to 12 months. Most participants (n = 41 [74.5%]) had a family history of HA, with confirmed maternal inheritance of the affected *F8* gene. Family history of FVIII inhibitors was reported for 7 participants (12.7%).

**Table 1.** **Participant demographics, baseline clinical characteristics, and medical history**

	Participants (N = 55)
**Age at informed consent, mo**	
Mean (SD)	5.0 (3.9)
Median (range)	4.0 (9 days to 11 months 30 days)
**Age group, n (%)**	
0-<3 mo	25 (45.5)
3-12 mo	30 (54.5)
Sex, n (%), male	55 (100)
**Ethnicity, n (%)**	
Hispanic or Latino	5 (9.1)
Not Hispanic or Latino	49 (89.1)
Unknown	1 (1.8)
**Race, n (%)**	
Asian	3 (5.5)
Black or African American	1 (1.8)
Native Hawaiian or other Pacific Islander	1 (1.8)
White	48 (87.3)
Unknown	2 (3.6)
**Weight at baseline, kg**	
Median (range)	7.1 (3.2-12.0)
**Mode of delivery, n (%)**	
Vaginal delivery (not assisted)	18 (32.7)
Vaginal delivery (assisted)	6 (10.9)
Planned cesarean delivery	30 (54.4)
Emergency cesarean delivery	1 (1.8)
**Family history of HA, n (%)**	41 (74.5)
Family history of FVIII inhibitors	7 (12.7)
**Prior treatment status, n (%)**	
MTP∗	30 (54.5)
PUP	25 (45.5)
**Hemophilia treatments received prior to first emicizumab dose**	
Participants with ≥1 treatment, n (%)	34 (61.8)
Total number of treatments, n	85
**Purpose of treatment, n (%)**	
Treatment for a bleed	30 (54.5)
Preventive dose before activity	4 (7.3)
Preventive dose for procedure/surgery	3 (5.5)
**Historical bleeding episodes prior to first emicizumab dose**	
Participants with ≥1 bleed, n (%)	36 (65.5)
Total number of bleeds, n	77
**Cause/type of bleed, n (%)**	
Spontaneous	25 (32.5)
Joint	8 (32.0)†
Muscle	6 (24.0)‡
Other	11 (44.0)§
Traumatic	19 (24.7)
Joint	0 (0.0)
Muscle	1 (5.3)‡
Other	18 (94.7)§
Procedural/surgical	33 (42.9)
Joint	0 (0.0)
Muscle	8 (24.2)‡
Other	25 (75.8)§

Age is calculated relative to the date when the informed consent form was signed.

∗ Defined as a participant with ≤5 EDs to hemophilia-related treatments containing FVIII, such as plasma-derived FVIII, recombinant FVIII, fresh/frozen plasma, cryoprecipitate, or whole blood products.

† Of the 8 total prestudy joint bleeds, 2 each occurred in the elbow and hip and 1 each occurred in the ankle, fingers/thumb, knee, and shoulder.

‡ Of the 15 total prestudy muscle bleeds, the majority (8 bleeds [n = 7 participants]) occurred in the thigh. HA diagnosis was known in 4 of these participants at the time of these bleeds in the thigh. Six of the 8 bleeds in the thigh were procedural bleeds: 3 for vaccination (n =3 participants); 3 for vitamin K administration (n = 2 participants; [in 1 participant, 1 bleed in the left thigh; in another participant, 1 bleed in the left thigh and 1 bleed in the right thigh]). The remaining 2 bleeds in the thigh were 2 spontaneous bleeds in the left thigh (n = 2 participants).

§ Of the 54 total other prestudy bleeds, 9 occurred in the sole/heel (all due to heel prick for metabolic tests [n = 6 participants], with 2 of 6 having received HA diagnosis at the time of these bleeds), 6 in the back of the hand or the mouth (HA diagnosis known in 5 of 6 cases [n = 4 participants] at the time of these bleeds), and the remainder were distributed across the rest of the body.

Thirty of the 55 participants (54.5%) were MTPs, and 25 (45.5%) were PUPs. MTPs had a median of 2 (range, 1-6) FVIII EDs. Before study, 1 participant received 6 doses of FVIII, of which 2 were administered on consecutive calendar days and considered as 2 EDs despite being administered within 24 hours. Before study entry, 34 of the 55 total participants (61.8%) had received a total of 85 administrations of factor-based therapies or antifibrinolytics, mostly for bleed treatment; 4 of these participants had received only antifibrinolytics and were, therefore, still classed as PUPs.

Approximately two-thirds of participants (36 of 55 [65.5%]) had already experienced ≥1 bleed (treated or untreated) before receiving emicizumab (Table 1; individual historical and on-study bleeding episodes can be found in supplemental Figure 1, available on the *Blood* website). The reporting period was variable across the 36 participants who had ≥1 bleed before receiving emicizumab, with the median age at the time of first historical treated or untreated bleed being 1 week (range, 0-49). Approximately one-third of the 77 prestudy bleeds (32.5%) were spontaneous, 24.7% were traumatic, and 42.9% procedural/surgical (including, but not limited to, procedures such as birth delivery method, vaccination, vitamin K administration, and heel-prick metabolic tests). Seven participants (12.7%) had experienced ≥1 joint bleed, and the age at the time of first joint bleed ranged from 14 to 34 weeks.

### Efficacy

Model-based ABRs were consistently low across bleeding end points (Table 2). The model-based ABR was 2.0 (95% CI, 1.49-2.66) for all bleeds, 0.4 (95% CI, 0.30-0.63) for treated bleeds, 0.0 (95% CI, 0.01-0.09) for treated joint bleeds, and 0.1 (95% CI, 0.02-0.12) for treated muscle bleeds. There were no treated spontaneous bleeds, because all treated bleeds were traumatic.

**Table 2.** **Bleeding outcomes: overall population**

	Participants (N = 55)
Median (range) follow-up,∗ wk	101.9 (52.6-119.7)
**Model-based ABR (95% CI)**	
All bleeds	2.0 (1.49-2.66)
Treated bleeds	0.4 (0.30-0.63)
Treated spontaneous bleeds	0.0†
Treated joint bleeds	0.0 (0.01-0.09)
**Calculated median ABR (IQR)**	
All bleeds	1.0 (0.53-2.93)
Treated bleeds	0.0 (0.00-0.81)
Treated spontaneous bleeds	0.0 (0.00-0.00)
Treated joint bleeds	0.0 (0.00-0.00)
**Participants with 0 bleeds, n (%)**	
Zero treated or untreated bleeds	9 (16.4)
Zero treated bleeds	30 (54.5)
Zero treated spontaneous bleeds	55 (100.0)
Zero treated joint bleeds	52 (94.5)
Participants with ≥1 bleed, n (%)	46 (83.6)
Total number of bleeds,‡ n	207
**Cause/type of bleed, n (%)**	
Spontaneous	18 (8.7)
Joint	0 (0.0)
Muscle	0 (0.0)
Other	18 (100.0)
Traumatic	182 (87.9)
Joint	4 (2.2)
Muscle	5 (2.7)
Other	173 (95.1)
Procedural/surgical§	7 (3.4)
Joint	0 (0.0)
Muscle	1 (14.3)
Other	6 (85.7)
Participants with ≥1 treated bleed, n (%)	25 (45.5)
Total number of treated bleeds, n	42
**Cause/type of treated bleed, n (%)**	
Traumatic	42 (100.0)
Joint	3 (7.1)
Muscle	5 (11.9)
Other	34 (81.0)

At time of the primary analysis, the median age of the participants was 29 months (min-max, 12-39).

IQR, interquartile range.

∗ The start of the efficacy period for each individual participant was defined as the day of the first emicizumab dose. The end of the efficacy period was defined as the date of the clinical cutoff or the date of withdrawal from the study period (ie, “open-label treatment” and “long-term follow-up” according to the eCRF), whichever was earlier.

† ABR could not be estimated via the negative binomial regression model because no treated spontaneous bleeds were observed in the study; as a result, a value of 0.0 is reported in the table instead.

‡ Two participants experienced >10 bleeds, none treated and none in a joint or muscle. One participant experienced 27 bleeds (19 traumatic and 8 spontaneous; 20 of 27 being nosebleeds), with the first bleed recorded at ∼11.6 months of age. The other participant experienced 21 bleeds (all traumatic), with the first bleed reported at ∼13.8 months of age.

§ Of the 7 procedural/surgical bleeds, 4 bleeds were reported in 1 participant and 1 bleed each was reported in 3 participants.

After a median duration of 101.9 weeks (range, 52.6-119.7) in the efficacy period, 54.5% of participants had 0 treated bleeds, 16.4% had 0 bleeds of any type, treated or untreated, and 94.5% had 0 treated joint bleeds.

Overall, 207 bleeds were reported in 46 participants (83.6%), 87.9% of which were traumatic. Two participants experienced >10 bleeds, but none were treated or had occurred in the joint or muscle: 27 bleeds in 1 participant (19 traumatic and 8 spontaneous) and 21 bleeds in the other (all traumatic). In total, 42 treated bleeds, all traumatic, were reported in 25 participants (45.5%). No participant experienced >3 treated bleeds.

One participant had his emicizumab dose up-titrated to 3 mg/kg once weekly per investigator request based on locally assessed decreasing emicizumab levels (confirmed retrospectively via central assessment to be 6.6 μg/mL at the lowest). This participant experienced 3 treated bleeds before up-titration start (day 374) and 2 untreated bleeds after up-titration until CCOD (328 days later); all were traumatic.

The median age at the time of the first on-study bleed was 53.0 weeks (range, 12-127; n = 46). At CCOD, only 4 participants (7.3%) had reported an on-study joint bleed (all traumatic), which occurred at an age of 29 to 124 weeks.

### Safety

At primary analysis, no ICH had occurred, and no new safety signals were identified, with no AEs leading to study discontinuation or treatment changes or withdrawal (Table 3). All participants experienced an AE, with 631 reported in total. Sixteen participants (29.1%) experienced a total of 30 serious AEs (SAEs), most of which were infant specific, including respiratory-related and head-injury events (detailed in Table 3 footnotes). In all cases, these were considered serious because of required or prolonged hospitalization. No SAEs were considered related to emicizumab.

**Table 3.** **Safety summary**

	Participants (N = 55)
Total number of AEs, n	631
**Participants with ≥1 AE, n (%)**	55 (100)
AE with fatal outcome	0 (0)
AE leading to withdrawal from treatment	0 (0)
AE leading to dose modification/interruption	0 (0)
Participants with ≥1 grade ≥3 AE, n (%)	17 (30.9)
**Participants with ≥1 treatment-related AE,**∗**n (%)**	9 (16.4)
Injection-site reaction, number of events	30
Total number of SAEs,† n	30
Participants with ≥1 SAE, n (%)	16 (29.1)
**AEs of special interest, n (%)**	1 (1.8)
Systemic hypersensitivity/anaphylactic/anaphylactoid reaction	1 (1.8)‡
Thromboembolic event	0 (0)
Thrombotic microangiopathy	0 (0)

∗ All treatment-related AEs were grade 1 local injection-site reactions.

† None of the SAEs were considered emicizumab related, and all were considered serious because of hospitalization. SAEs included fall (n = 4); head injury (n = 4); bronchiolitis, bronchitis, pneumonia, tonsillitis, mouth hemorrhage, tongue hemorrhage (n = 2 for each); and ear infection, laryngitis, upper respiratory tract infection, urinary tract infection, viral infection, eyelid contusion, postprocedural fever (liver biopsy because of fluctuating liver enzymes assessed locally; serology test results for Epstein-Barr virus, cytomegalovirus, human herpesvirus 6 and hepatitis A, B, and C were negative, and no liver pathology was found), postprocedural hemorrhage (tonsillectomy), skin laceration, and tongue injury (n = 1 for each).

‡ One anaphylactic reaction due to an egg allergy was reported in 1 participant, considered not related to emicizumab.

Thirty emicizumab-related AEs occurred in 9 participants (16.4%); all grade 1 injection-site reactions. One grade 2 anaphylactic reaction was reported in 1 participant (1.8%). This event resolved, was confirmed to be due to egg allergy, and deemed unrelated to emicizumab. No thromboembolic events or thrombotic microangiopathies were reported.

### PKs

**Figure 2.**
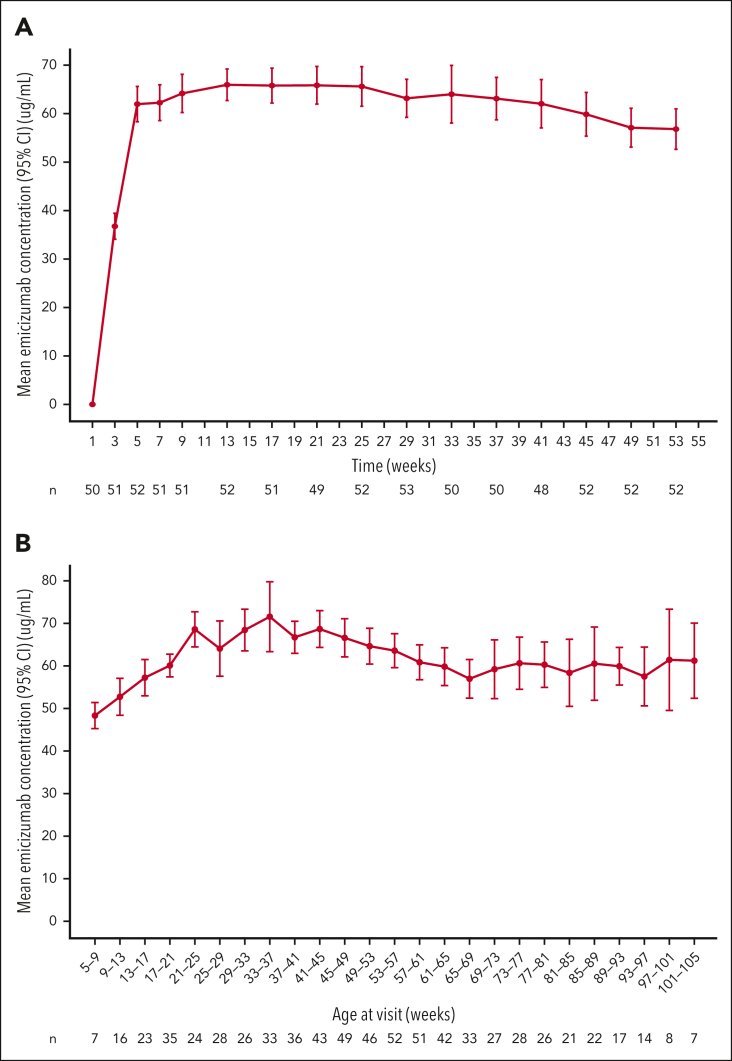
**Pharmacokinetics of emicizumab prophylaxis.** Mean (95% CI) emicizumab trough concentration at visit (A) over time and (B) based on age at visit during the maintenance period. For the participant whose dose was up-titrated, only data before up-titration are included. For the analysis according to the age, only samples from week 5 onwards (maintenance period) were considered.

Mean trough concentrations of emicizumab increased during loading, reaching 62.0 (95% CI, 58.3-65.6) μg/mL at week 5 (Figure 2A). Thereafter, steady-state concentrations were sustained at 57 to 66 μg/mL. Mean steady-state trough concentrations increased slightly with age until participants reached age ∼6 months, at which trough concentrations were maintained at ≥60 μg/mL (Figure 2B).

### Biomarkers

Mean FIX and FX antigen concentrations were not affected by emicizumab treatment (supplemental Figures 2A and 3A) but did increase with age (supplemental Figures 2B and 3B). aPTT was shortened to within reference range by day 15 for most participants, the first time point at which blood samples were obtained, because of limitations on sampling in infants (supplemental Figure 4A; results as per the age in supplemental Figure 4B).

Mean TG peak height increased during loading and was maintained between 67 and 88 nmol/L thereafter (supplemental Figure 5A; results as per the age in supplemental Figure 5B).

Mean FVIII-like activity increased from 1.0 U/dL (standard deviation [SD], 0.9) at baseline (n = 48) to 22.5 U/dL (SD, 6.1) at week 5 (n = 50) and was sustained between 21 and 26 U/dL thereafter (supplemental Figure 6A; results as per the age in supplemental Figure 6B).

### Immunogenicity

All 55 participants were evaluable for immunogenicity; none tested positive for ADAs to emicizumab.

On study, 28 participants (50.9%) received a total of 139 administrations of factor-based therapy, including FVIII in all 28 participants and recombinant activated FVII in 1 participant after the development of FVIII inhibitors. Median on-study FVIII ED(s) was 1.0 (range, 0-10), with a mean of 1.8 doses (SD, 3.3). On-study FVIII EDs were similar between PUPs (median in 14 of 25 participants, 1.0; range, 0-10) and MTPs (median in 14 of 30 participants, 0.0; range, 0-10). At CCOD, 11 of 25 PUPs (44.0%) and 16 of 30 MTPs (53.3%) had not reported an on-study FVIII ED. The median cumulative dose of FVIII received per participant was 1250 IU (range, 250-15 600 IU). In 25 participants (45.5%), factor-based therapy was received for the treatment of a bleed; in 4 participants (7.3%) as additional prophylaxis before activity (no information about the activity was available); and in 5 (9.1%) as additional prophylaxis for a procedure/surgery; some participants received factor-based therapy for >1 of these reasons.

During the study, 24 participants (43.6%) were tested for FVIII inhibitors after FVIII exposure, with 2 (3.6%) showing positive results. These 2 participants were both PUPs aged 0 to <3 months at informed consent, with confirmed maternal inheritance of the affected *F8* gene, and 1 of the 2 participants had a reported family history of inhibitors. One PUP was confirmed for presence of inhibitors on day 603 (6.9 chromogenic BU [CBU]/mL) and on day 681 (1.5 CBU/mL) after 3 nonconsecutive standard half-life FVIII EDs for bleed treatment. The other tested positive for inhibitors (28.4 CBU/mL) on day 428, after 10 nonconsecutive extended half-life FVIII EDs related to bleed treatment and surgical procedures; inhibitors were confirmed on day 532 (9.0 CBU/mL). Narratives can be found in the supplemental Data.

## Discussion

Early prophylaxis in infants with HA is important to protect long-term joint function and reduce potentially life-threatening bleeds such as ICH, which remains a significant concern in this population.^5^ The subcutaneous nature of emicizumab administration makes prophylaxis initiation practicable at a very young age. Primary analysis of the HAVEN 7 trial indicates the efficacy and favorable safety profile of emicizumab prophylaxis for infants aged ≤12 months with severe HA without FVIII inhibitors. Results support the guidance of the World Federation of Hemophilia and the National Bleeding Disorders Foundation’s Medical and Scientific Advisory Council, both of which indicate that infants should be considered for prophylaxis with emicizumab any time after birth, given the increased risk of ICH in this population.^1^^,^^37^ In addition, at study entry, 65.5% of participants had already experienced ≥1 bleed, and 12.7% had ≥1 joint bleed, with the age at first bleed and first joint bleed ranging from 0 to 49 weeks and 14 to 34 weeks, respectively. These data support the need for very early prophylaxis, before age 3 months.

After a median efficacy period of 101.9 weeks, no cases of ICH were recorded despite 4 SAEs of head injury. These data suggest that emicizumab prophylaxis may reduce the risk of ICH, given the known incidence of ICH and risk continuum^5^; however, it should be noted that the study was not powered to demonstrate this. Model-based ABR was 0.4 (95% CI, 0.30-0.63) for treated bleeds (all traumatic), consistent with results from HAVEN 2; children in HAVEN 2 receiving emicizumab 1.5 mg/kg once weekly (n = 65, including 8 infants aged <2 years) had a model-based treated bleed ABR of 0.3 (95% CI, 0.17-0.50).^19^ The proportion of participants with zero treated bleeds in HAVEN 7 was also consistent with findings from other emicizumab clinical trials when accounting for variable follow-up periods. The HAVEN 7 interim analysis had a median exposure duration of 42.1 weeks (range, 1-60). As expected, a higher proportion of participants (42/54 [77.8%]) had 0 treated bleeds after the shorter follow-up time at interim analysis than at primary analysis (54.5%),^38^ consistent with the 77% of participants reported in the 1.5 mg/kg once weekly group of HAVEN 2 (median efficacy period, 57.6 weeks; range, 17.9-92.6 weeks), 100% of participants aged <2 years in HOHOEMI (n = 3; efficacy period, 24.1-38.4 weeks), and adults and adolescents in other HAVEN trials with similar follow-up periods.^18^, ^19^, ^20^, ^21^, ^22^, ^23^^,^^25^ In the real-world setting, Barg et al reported no joint or spontaneous bleeds over a median of 36 weeks for 11 infants with HA receiving emicizumab with a median age of 26 months at study entry,^26^ Garcia and Zia reported no joint or spontaneous bleeds in 3 infants aged <3 years after receiving emicizumab for 9 to 15 months,^27^ and Mason and Young reported no bleeds in 4 infants receiving emicizumab aged <2 years after a median follow-up of 12 months.^28^

At CCOD, only 4 participants had experienced an on-study joint bleed, with age range at the time being 29 to 124 weeks. Joint bleeds typically occur at an older age than other bleed types,^38^ and results of the 7-year follow-up will provide data on long-term joint health in the setting of earliest initiation of emicizumab prophylaxis.

In line with the safety profile of emicizumab in clinical trials,^18^, ^19^, ^20^, ^21^, ^22^, ^23^ no new safety signals were found at primary analysis of HAVEN 7, and all emicizumab-related AEs were grade 1 injection-site reactions. Most SAEs in HAVEN 7 were infant specific, including respiratory-related AEs such as bronchiolitis leading to hospitalization and head injuries after which the infant was brought to hospital for observation, with imaging in some cases to confirm the absence of ICH.

Mean steady-state emicizumab concentrations were 57 to 66 μg/mL at CCOD, above those reported previously in older people with HA in the phase 3 HAVEN 1 to 4 trials (46.7 μg/mL; SD, 14.9 μg/mL, for participants receiving emicizumab 3 mg/kg once every 2 weeks).^24^ No confirmed explanation has been identified for these higher concentrations in comparison with other HAVEN trials. Age is not believed to be a factor, because the emicizumab concentrations reported here are numerically higher than in the data from HAVEN 2 and HOHOEMI, which included children with HA of similar ages,^19^^,^^25^ whereas a population PK model suggested lower exposure at age <1 year.^30^ Emicizumab injection site might have played a role, because during the first 5 weekly administrations in HAVEN 7 when emicizumab injection sites were recorded, 80% of administrations were in the thigh, and a numerical trend for higher exposure after thigh injection than after abdomen or upper arm has been observed in a study of the relative bioavailability of emicizumab across injection sites.^39^ In contrast to the higher exposure, analysis of coagulation biomarkers in HAVEN 7 indicates that, at the same emicizumab concentration, TG and FVIII-like activity are somewhat lower in infants than in older populations^40^; therefore, the hemostatic effect of emicizumab is expected to be correspondingly lower.

Similar to our observation of increasing FIX and FX protein levels up to age 9 months in participants, FIX and FX activities in healthy infants have been shown to increase during development from low levels in infancy to reach near adult levels at 6 months.^41^ Lower FIX and FX plasma levels in the youngest participants might have had an impact on the PD results. However, increases in FVIII-like activity and TG were seen with steady-state emicizumab for all age groups. Irrespective of considerations regarding the developing coagulation system at such young ages, treatment of infants in HAVEN 7 with the approved dose of emicizumab was well tolerated and efficacious.

No participant in HAVEN 7 had tested positive for ADAs at CCOD. This reflects the low immunogenicity rate for emicizumab reported in a pooled analysis of the phase 3 clinical trials HAVEN 1 to 5, HOHOEMI, and STASEY, across which 5.1% of participants developed ADAs, including 0.6% for whom ADAs were associated with a decrease in emicizumab exposure.^34^ In HAVEN 7, a total of 24 participants were tested for FVIII inhibitors after at least 3 EDs or 2 consecutive doses of FVIII; 2 participants (3.6% of the trial population and 8.3% of those tested), both PUPs, tested positive for confirmed de novo FVIII inhibitors. Because 28 of 55 of the trial population (∼50%) received FVIII treatment on study (with a median of 1 ED) and only 24 of 55 were tested for FVIII inhibitors, many participants are still in the ED risk period for inhibitor development. The long-term follow-up will provide further data on the impact of emicizumab on rate and timing of FVIII inhibitor development.

The HAVEN 7 trial has limitations to note. It is open-label and single-arm, and all analyses are descriptive because no formal hypothesis testing was planned. In addition, almost half of the participants were aged <3 months at the time of informed consent, so comparison of bleed history since birth is limited, although two-thirds had already experienced ≥1 treated or untreated bleed at baseline. Moreover, the relatively short follow-up time limits accurate assessment of the effect of emicizumab on joint health and time to first bleed, although the long-term follow-up period will offer further insight. Finally, despite efforts to recruit female infants, none were enrolled due to the low frequency of severe HA in females and later diagnosis than males.^42^

This primary analysis of HAVEN 7 indicates that emicizumab is efficacious and well tolerated in infants with severe HA without FVIII inhibitors at a currently approved dose. No participant developed ADAs. Future analyses of HAVEN 7 will describe the natural history of children with HA who initiate emicizumab prophylaxis soon after birth, including, but not limited to, safety and joint health outcomes, over the 7-year follow-up period.

Conflict-of-interest disclosure: S.W.P. is a member of the scientific advisory board for GeneVentiv and Equilibra Bioscience and has received grants or contracts from Siemens; consulting fees from Apcintex, ASC Therapeutics, Bayer, BioMarin, CSL Behring, HEMA Biologics, Freeline, LFB Group, Novo Nordisk, Pfizer, Regeneron/Intellia, Genentech, Inc./F. Hoffmann-La Roche Ltd, Sanofi, Takeda, Spark Therapeutics, and uniQure. P.C. is a member on an entity’s board of directors for the HAVEN 7 trial steering committee and a member of the UK Haemophilia Centre Doctors’ Organization, which has received a research grant from F. Hoffmann-La Roche Ltd. C.D. is an employee of F. Hoffmann-La Roche Ltd. G.K. has received grants/research support funding from Binational Science Foundation, Pfizer, F. Hoffmann-La Roche Ltd, Tel Aviv University, and Sheba research authorities; consulting fees from ASC Therapeutics, Bayer, BioMarin, Novo Nordisk, Pfizer, F. Hoffmann-La Roche Ltd, Sobi, Sanofi-Genzyme, Takeda, and uniQure; honoraria from Bayer, BioMarin, Bio Products Laboratory, CSL Behring, Pfizer, Novo Nordisk, F. Hoffmann-La Roche Ltd, Sanofi-Genzyme, and Spark Therapeutics; participated on a data safety monitoring board or advisory board for ASC Therapeutics, BioMarin, Pfizer, Novo Nordisk, uniQure, F. Hoffmann-La Roche Ltd, Sanofi-Genzyme, Sobi, and Spark Therapeutics; and has a leadership role in PedNet Research foundation. C.S. and M.B. are employees and stockholders of F. Hoffmann-La Roche Ltd. V.J.-Y. has received grants or contracts from F. Hoffmann-La Roche Ltd, Novo Nordisk, Sobi, Takeda, Grifols, Bayer, Pfizer, Octapharma, and CSL Behring; consulting fees from F. Hoffmann-La Roche Ltd, Novo Nordisk, Sanofi, Sobi, Takeda, Grifols, Bayer, Pfizer, Spark Therapeutics, BioMarin, Octapharma, and CSL Behring; and honoraria from F. Hoffmann-La Roche Ltd, Novo Nordisk, Sanofi, Sobi, Takeda, Grifols, Bayer, Pfizer, Spark Therapeutics, BioMarin, Octapharma, and CSL Behring. F.P. has received speaker fees for participating in advisory boards from Sanofi, Sobi, Takeda, F. Hoffmann-La Roche Ltd, and BioMarin; and educational meeting fees from Grifols and F. Hoffmann-La Roche Ltd. G.Y. has received grants or contracts from Genentech, Inc., Grifols, and Takeda; royalties or licenses from Viatris; consulting fees from Bayer, BioMarin, CSL Behring, Genentech, Inc./F. Hoffmann-La Roche Ltd, LFB Group, Novo Nordisk, Pfizer, Sanofi, Spark Therapeutics, and Takeda; honoraria from Bayer, BioMarin, CSL Behring, Genentech, Inc./F. Hoffmann-La Roche Ltd, Novo Nordisk, Pfizer, Sanofi, Spark Therapeutics, and Takeda; and speakers bureau fees from BioMarin, Genentech, Inc., Hema Biologics, Sanofi, and Spark Therapeutics. J.O. has received research funding from Bayer, Biotest, CSL Behring, Octapharma, Pfizer, Swedish Orphan Biovitrum, and Takeda; consultancy, speakers bureau fees, honoraria, scientific advisory board fees, and travel expenses from Bayer, Biogen Idec, BioMarin, Biotest, Chugai Pharmaceutical Co., Ltd, CSL Behring, Freeline, Grifols, LFB Group, Novo Nordisk, Octapharma, Pfizer, F. Hoffmann-La Roche Ltd, Sanofi, Spark Therapeutics, Swedish Orphan Biovitrum, and Takeda. M.E.M. has received consulting fees from Bayer, CSL Behring, Novo Nordisk, F. Hoffmann-La Roche Ltd, Octapharma, Pfizer, Sanofi, Sobi, Kedrion, Grifols, BioMarin, Catalyst, uniQure, and LFB Group; honoraria from Bayer, CSL Behring, Novo Nordisk, F. Hoffmann-La Roche Ltd, Octapharma, Pfizer, Sobi, Kedrion, Grifols, BioMarin, and Spark Therapeutics. K.K. has received consulting and honoraria fees from F. Hoffmann-La Roche Ltd, Novo Nordisk, Takeda, and Pfizer; and research funding from F. Hoffmann-La Roche Ltd and Pfizer. S.D. is an employee of Genentech, Inc. A.K., M.N., and M.L. are employees and have stock ownership in F. Hoffmann-La Roche Ltd. T.C is an employee of Spark Therapeutics, which is part of the Roche group and holds stock in F. Hoffmann-La Roche Ltd. The institution of K.F. has received unrestricted research grants from CSL Behring, Sobi, and Novo Nordisk; and consultancy fees from F. Hoffmann-La Roche Ltd, Sanofi, Sobi, and Novo Nordisk.
