# Supplementary material for: Emicizumab prophylaxis in infants with hemophilia A (HAVEN 7): primary analysis of a phase 3b open-label trial
Source: Blood. 2023 Dec 23;143(14):1355–64. doi: 10.1182/blood.2023021832 (PMC11033591; doi:10.1182/blood.2023021832)
Supplement: Supplemental Methods, Table and Figures [file BLOOD_BLD-2023-021832-mmc1.pdf]

Supplemental data

Inclusion and exclusion criteria

Full inclusion criteria

- Eligible participants were infants from birth to ≤12 months of age weighing ≥3 kg at the time of informed consent.
  - Written informed consent was obtained from a parent or caregiver prior to any trial-specific assessments or procedures being performed.
  - Participants had a diagnosis of severe congenital hemophilia A (HA) (intrinsic factor [F]VIII level ≤1%), a negative test for FVIII inhibitors (<0.6 Bethesda Unit [BU]/mL) locally assessed during the 2-week screening period, and no history of documented FVIII inhibitors, FVIII drug-elimination half-life <6 hours, or FVIII recovery <66%.
  - Participants were previously untreated or minimally treated (i.e., up to 5 days of exposure with hemophilia-related treatments containing FVIII such as plasma-derived FVIII, recombinant FVIII, fresh frozen plasma, cryoprecipitate, or whole blood products).
  - Receipt of vitamin K prophylaxis was mandatory if required by local standard practice.
  - Participants required documentation of the details of bleeding episodes and hemophilia-related treatments since birth, and had no evidence of active intracranial hemorrhage (ICH), as confirmed by a negative cranial ultrasound at screening.
  - Participants had adequate hematologic function (defined as platelet count ≥100,000/μL [≥100 × 10^9 cells/L] and hemoglobin ≥8 g/dL [4.97 mmol/L]), and adequate hepatic function (defined as total bilirubin ≤1.5 × the age-specific upper limit of normal [ULN]; excluding infants with Gilbert syndrome or benign neonatal hyperbilirubinemia because of breastfeeding; and both aspartate aminotransferase and alanine aminotransferase ≤3 × the age-specific ULN) at screening, and adequate renal function (defined as serum creatinine ≤1.5 × the age-specific ULN; if the serum creatinine was ≥1.5 × the age-specific ULN, creatinine clearance by Schwartz estimation was required to be >70 mL/min/1.73 m^2).

Full exclusion criteria

- Infants who met any of the following criteria were excluded from trial entry: inherited or acquired bleeding disorder other than severe HA; use of systemic immunomodulators at enrollment or planned use during the trial; prior use of emicizumab prophylaxis; or receipt of an investigational drug to treat or reduce the risk of hemophilic bleeds within five drug-elimination half-lives of last drug administration, a non-hemophilia-related investigational drug within the last 30 days or five drug-elimination half-lives, whichever is shorter, or a concurrent investigational drug.
- Exclusion criteria also included current active severe bleeds (such as ICH; participants from birth to <3 months of age must not demonstrate evidence of active ICH at cranial ultrasound performed at screening); planned surgery during the trial (excluding minor procedures such as circumcision or central venous access device placement); a history of clinically significant hypersensitivity associated with monoclonal antibody therapies or components of the emicizumab injection; being at high risk for thrombotic microangiopathies (TMA) in the investigator's judgement; previous or current treatment for thromboembolic disease (except previous catheter-associated thrombosis in infants for whom anti-thrombotic treatment is not currently ongoing) or signs of thromboembolic disease; any hereditary or acquired maternal condition that may predispose the infant to thrombotic events; other diseases that may increase risk of bleeding or thrombosis; known infection with human immunodeficiency virus (HIV), hepatitis B virus or hepatitis C virus; serious infection requiring antibiotics or antiviral treatments within 14 days prior to screening; and any concurrent disease, treatment, abnormality in clinical laboratory tests, vital sign measurements, or physical examination findings that could interfere with the trial or that would, in the opinion of the investigator or Sponsor, preclude the infant's safe participation in and completion of the trial or interpretation of the trial results.
- Parents and caregivers had to be willing to allow receipt of blood or blood products, or any standard-of-care treatment for a life-threatening condition.
- Any other medical, social or other condition that may prevent adequate compliance with the trial protocol in the opinion of the investigator would also result in exclusion.

Definitions of bleed endpoints

Bleed endpoints assessed in the HAVEN 7 trial include treated bleed rate, all bleed rate, treated spontaneous bleed rate, and treated joint bleed rate. Bleeds by cause, type and location were recorded as an exploratory bleed-related endpoint.

For the purpose of the efficacy analyses, a standardized definition of bleed, adapted from criteria defined by the Subcommittee on Standards and Criteria, FVIII/X subcommittee of the International Society of Thrombosis and Hemostasis,[3] was used in this trial as follows:

- An event was considered a treated bleed if coagulation factors were administered to treat signs or symptoms of bleeding (e.g., pain, swelling). An additional definition of all reported bleeds (irrespective of treatment with coagulation factors) was applied for a separate analysis.
- A new bleed was defined as a bleed occurring >72 hours after the last treatment for the original bleed. Any symptoms of bleeding that occurred ≤72 hours after the last treatment in the same location were considered the same bleed.
- Any injection administered to treat the bleed >72 hours after the preceding injection, was considered the first injection to treat a new bleed at the same location.
- Any bleed at a different location was considered a separate bleed regardless of time from the last injection.

Definitions of bleed causes

The assessment of a bleed was separated into spontaneous bleeds, traumatic bleeds, and bleeds related to procedure/surgery. Both spontaneous bleeds (i.e., the occurrence of hemorrhage for which the parents/caregivers cannot identify a reason) and traumatic bleeds (i.e., hemorrhage occurring secondary to an event such as trauma, “strenuous” activity, or “overuse”) were recorded.

- Spontaneous bleeds: Bleeds were classified as spontaneous if the parents/caregivers recorded a bleed when there was no known contributing factor such as definite trauma, antecedent “strenuous” activity, or “overuse.” The determination of what constitutes “strenuous” or “overuse” was at the discretion of the parents/caregivers.
- Traumatic bleeds: Bleeds were classified as traumatic if parents/caregivers recorded a bleed when there was a known or believed reason for the bleed.
- Bleeds related to procedure/surgery: for example, hematomas resulting from any surgery or invasive procedure (e.g., tooth extractions, venipuncture, or subcutaneous

study drug administrations), or invasive diagnostic procedures (e.g., lumbar puncture, arterial blood gas determination, or endoscopy with biopsy) were considered a bleed related to procedure/surgery. Such bleeds were not associated with any trauma except the procedure/surgery in question.

Definition of bleed types

Bleed types were defined as follows:

- Target joints: defined as a major joint (e.g., hip, elbow, wrist, shoulder, knee, or ankle) into which repeated bleeds occur (frequency of ≥3 bleeds into the same joint over a 24-week period)
- Joint bleeds: defined as bleeds with bleed type 'joint bleed' reported on the Bleed and Medication Questionnaire (eBMQ) with at least one of the following symptoms:
    - Increasing swelling or warmth of the skin over the joint
    - Increasing pain
    - Progressive loss of range of motion or difficulty in using the limb compared with baseline
- Muscle bleeds (sites per the eBMQ)
- Other (sites per the eBMQ)

Assessments and data collection

Participant discontinuation from the trial

Reasons for participant discontinuation from the trial might include but are not limited to: withdrawal of consent by parent or caregiver, trial termination or site closure, adverse event, unacceptable toxicity with study treatment, participant's requirement for immune tolerance induction therapy, loss to follow-up, or participant non-compliance (defined as failure to comply with protocol requirements as determined by the investigator or Sponsor).

Blood collection

EDTA and citrate plasma were collected as previously described,[34] with total blood collection volumes within the limits of European Commission guidelines on Ethical Considerations for Clinical Trials on Medicinal Products Conducted with the Paediatric Population or institutional guidelines.

Results

Subgroup analysis by age at informed consent

In a subgroup analysis by age at time of informed consent, 13/25 (52.0%) participants aged 0–<3 months and 12/30 (40.0%) participants aged 3–12 months experienced ≥1 treated bleed (**Table S1**). Median (min, max) treatment duration was 76.1 (52, 112) weeks for participants aged 0–<3 months, and 140.1 (52, 118) weeks for participants aged 3–12 months. In both age groups, most treated bleeds (81.0%, 17/21 bleeds in both group) were classed as 'other' bleeds (not joint or muscle). Other bleed outcomes were consistent across age groups. However, the proportion of spontaneous all bleeds was 3.4% (2/59 bleeds) in participants aged 0–<3 months at informed consent, versus 10.8% (16/148 bleeds) in participants aged 3–12 months, as expected by motor development in the slightly older age group. Further detail of motor development on bleeding outcomes is inferred in **Figure S1**.

Participant narratives for the two participants who developed FVIII inhibitors

The first participant, a PUP aged 0–<3 months at informed consent, with large *F8* deletion (FVIII genotype reported at baseline), had no reported family history of inhibitors. This participant was confirmed to have inhibitors (6.9 [CBU]/mL, on day 603; 1.5 CBU/mL on day 681) following three non-consecutive FVIII EDs, receiving five doses of standard half-life FVIII (500 IU; two doses on day 333, two doses on day 404, one dose on day 405) for the treatment of two traumatic mouth bleeds.

The second participant was a PUP aged 0–<3 months at informed consent, with intron 22 inversion (FVIII genotype reported at baseline), and had a reported family history of inhibitors. On day 279, the participant experienced a traumatic mouth bleed (treated with 350 IU extended half-life FVIII) and tested negative for FVIII inhibitors (0 CBU/mL, locally assessed). On day 414, the participant underwent adenotonsillectomy, receiving seven non-consecutive EDs of preventative extended half-life FVIII until day 422, ranging from 500 IU and 2550 IU on day 414 to 1000 IU on day 422. On day 425, the tonsillectomy procedure was resumed, which was accompanied by a post-procedural bleed treated with two EDs of extended half-life FVIII (day 425: 500 IU, 1200 IU, and 250 IU; day 426: 250 IU) and recombinant activated FVII (days 427–434). The participant tested positive for inhibitors on day 427 (5.9 CBU/mL, locally assessed) and day 428 (28.4 CBU/mL, centrally assessed). FVIII inhibitors were confirmed on day 532 (9.0 CBU/mL, centrally assessed).

These two participants had confirmed maternal inheritance of the affected *F8* gene, and one of the two had a reported family history of inhibitors. Per protocol, immune tolerance

induction was not provided to either participant. Emicizumab dosing was not modified or interrupted at any time.

Tables

Table S1. Bleeds during emicizumab prophylaxis by age at time of informed consent

	**Age at time of informed consent, months**	
	**0–<3 (n = 25)**	**3–12 (n = 30)**
**Median (min, max) efficacy period,* weeks**	76.6 (52.6, 114.0)	104.4 (53.4, 119.7)
**Participants with ≥1 bleed, n (%)**	19 (76.0)	27 (90.0)
**Total number of bleeds, n**	59	148
**Cause/type of bleed, n (%)**		
**Spontaneous**	**2 (3.4)**	**16 (10.8)**
Joint	0 (0.0)	0 (0.0)
Muscle	0 (0.0)	0 (0.0)
Other	2 (100.0)	16 (100.0)
**Traumatic**	**56 (94.9)**	**126 (85.1)**
Joint	1 (1.8)	3 (2.4)
Muscle	3 (5.4)	2 (1.6)
Other	52 (92.9)	121 (96.0)
**Procedural/surgical**	**1 (1.7)**	**6 (4.1)**
Joint	0 (0.0)	0 (0.0)
Muscle	0 (0.0)	1 (16.7)
Other	1 (100.0)	5 (83.3)
**Participants with ≥1 treated bleed, n (%)**	13 (52.0)	12 (40.0)
**Total number of treated bleeds, n**	21	21
**Cause/type of treated bleed, n (%)**		
**Traumatic**	**21 (100.0)**	**21 (100.0)**
Joint	1 (4.8)	2 (9.5)
Muscle	3 (14.3)	2 (9.5)
Other	17 (81.0)	17 (81.0)

*The start of the efficacy period for each individual patient is defined as the day of the first emicizumab dose. The end of the efficacy period is defined as the date of the clinical cut-off or the date of withdrawal from the study period (i.e., ‘Open Label Treatment’ and ‘Long-term Follow-up’ according to electronic Case Report Form), whichever is earlier.

Figures

Figure S1. Individual historical and on-study bleeding episodes by age at time of informed consent of (A) 0–<3 months and (B) 3–12 months

A

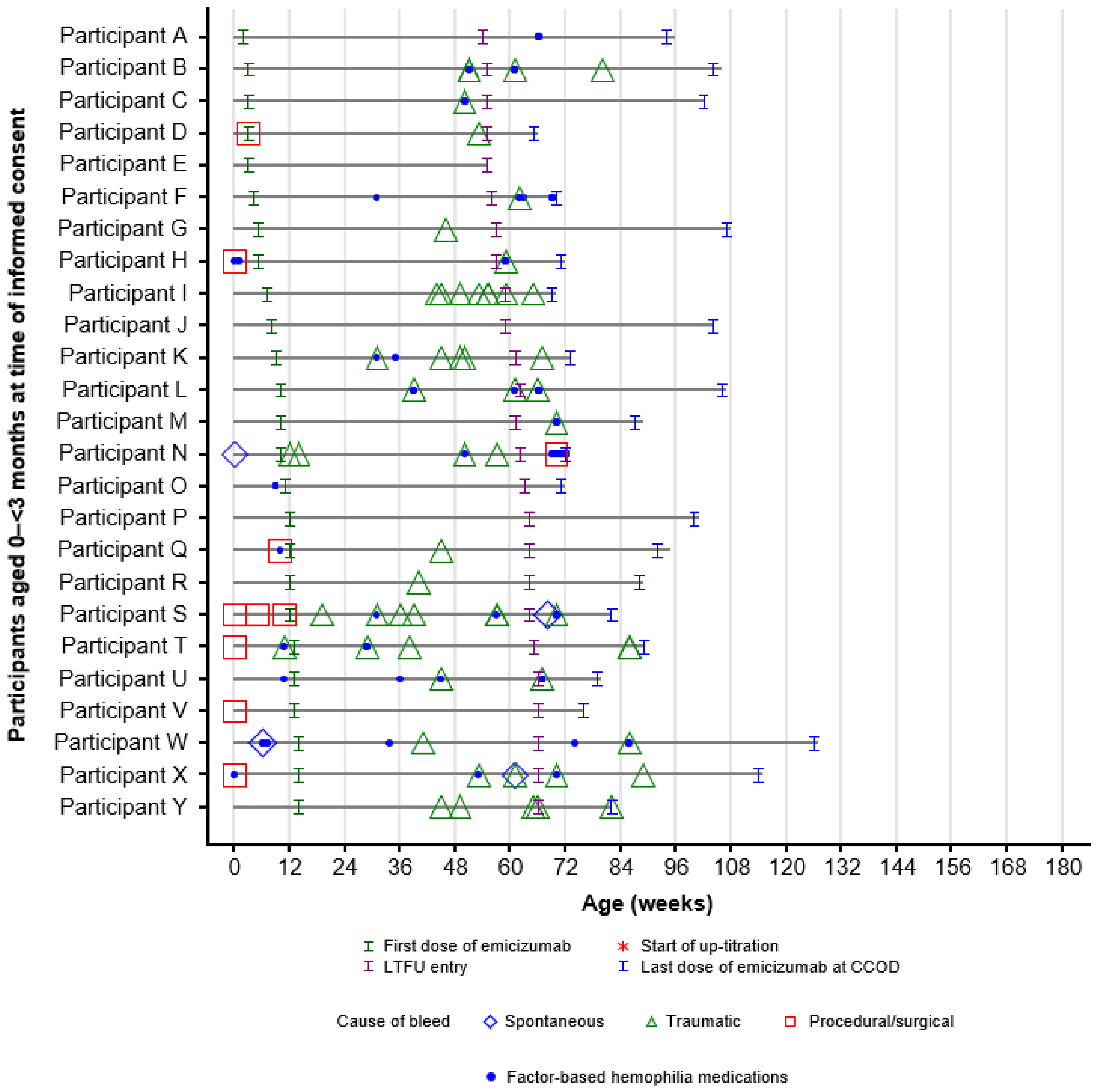

B

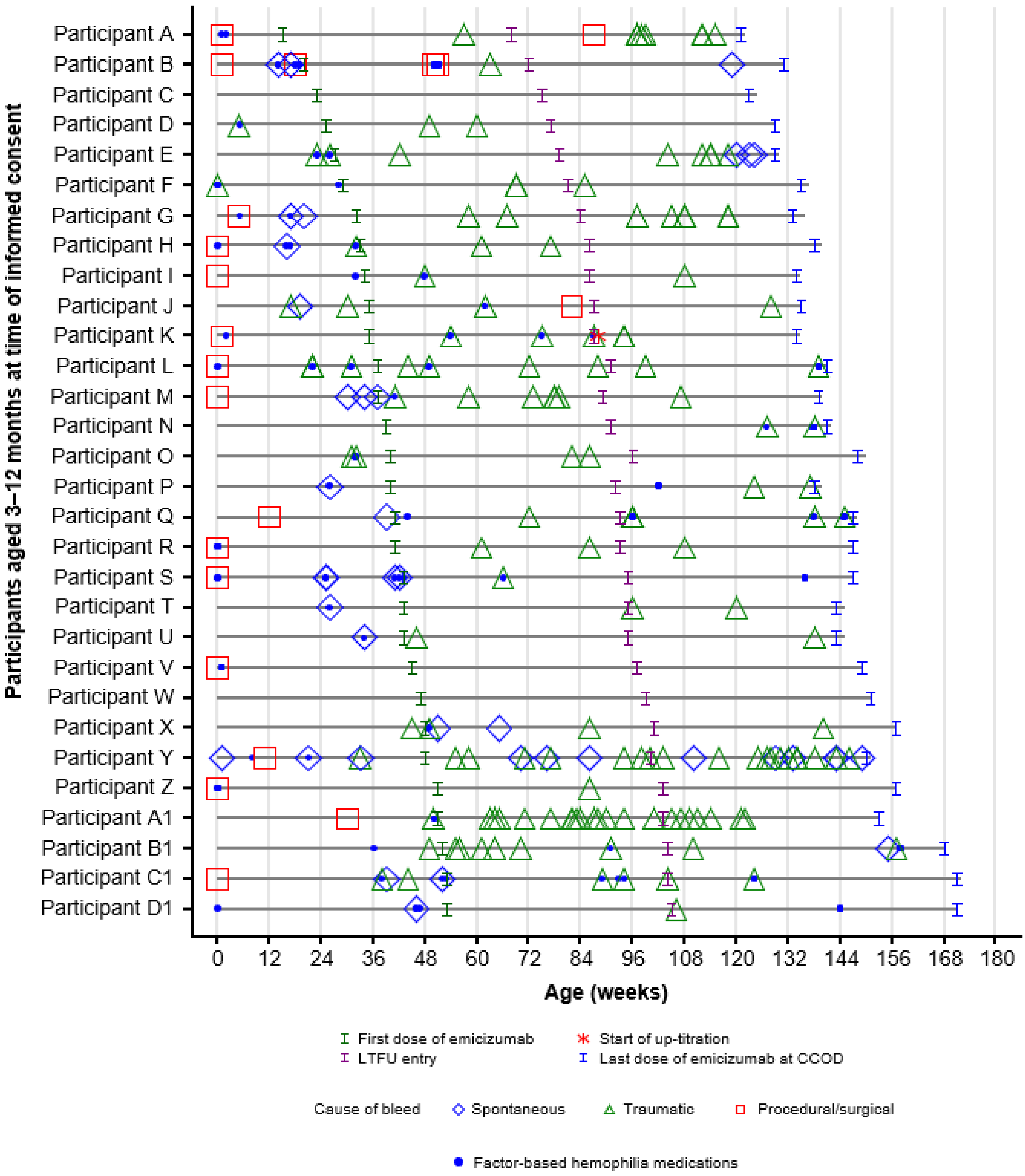

CCOD, clinical cut-off date; LTFU, long-term follow-up.

Figure S2. Mean and individual FIX concentration at visit (A) over time and (B) by age at visit during the maintenance period

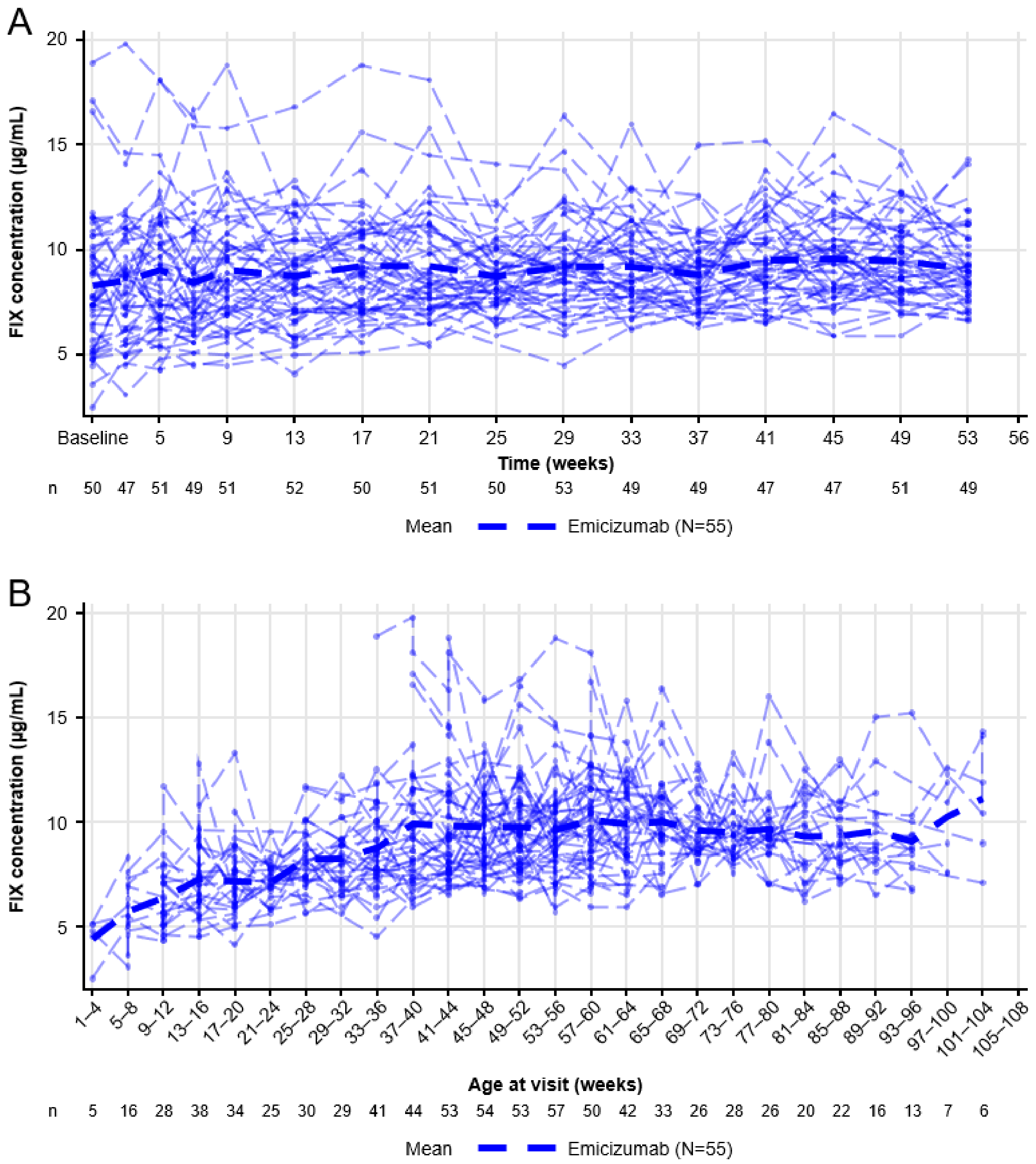

For the participant whose dose was up-titrated, only data before up-titration are included. For the analysis of FIX concentrations by age, all samples were considered. FIX, factor IX.

Figure S3. Mean and individual FX concentration (A) over time and (B) by age at visit during the maintenance period

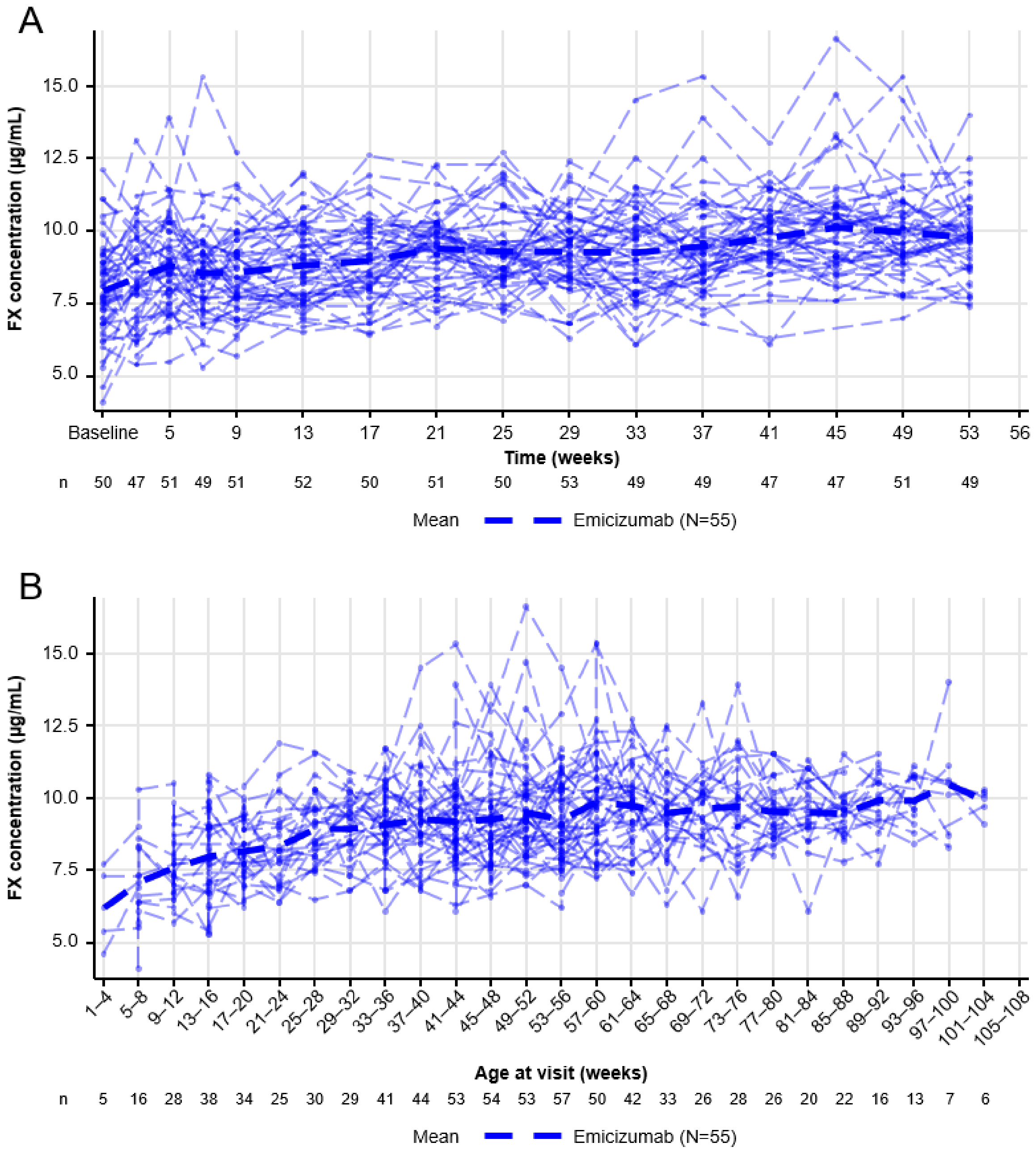

For the participant whose dose was up-titrated, only data before up-titration are included. For the analysis of FX concentrations by age, all samples were considered. FX, factor X.

Figure S4. Mean and individual aPTT (A) over time and (B) by age at visit during the maintenance period

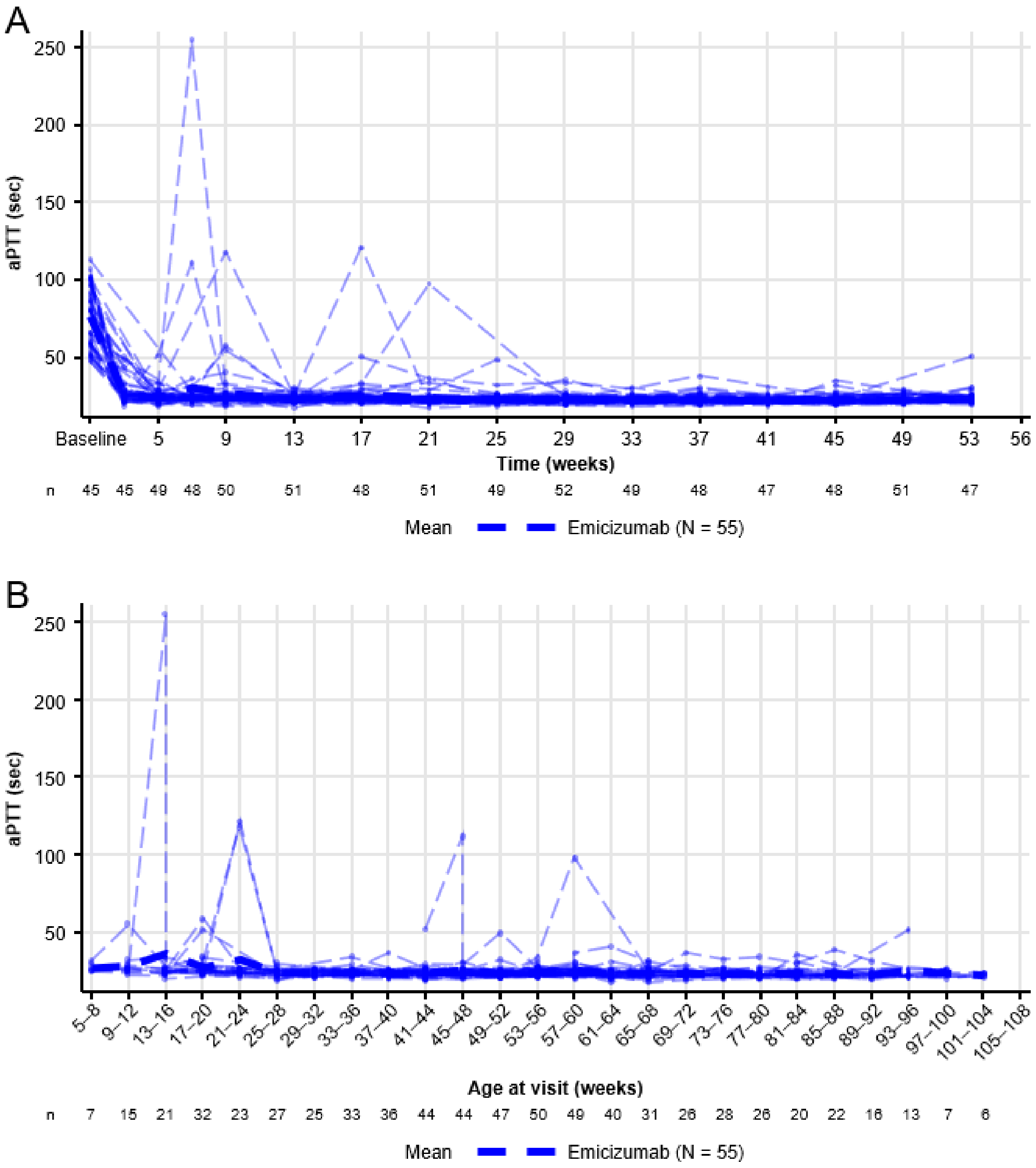

For the participant whose dose was up-titrated, only data before up-titration are included. For the analysis of aPTT by age, only samples from week 5 onwards (maintenance period) were considered. Values of aPTT reported as >255 seconds are replaced with 255 (ULOQ).

aPTT, activated partial thromboplastin time; ULOQ, upper limit of quantification.

Figure S5. Mean and individual TG peak height (A) over time and (B) by age at visit during the maintenance period

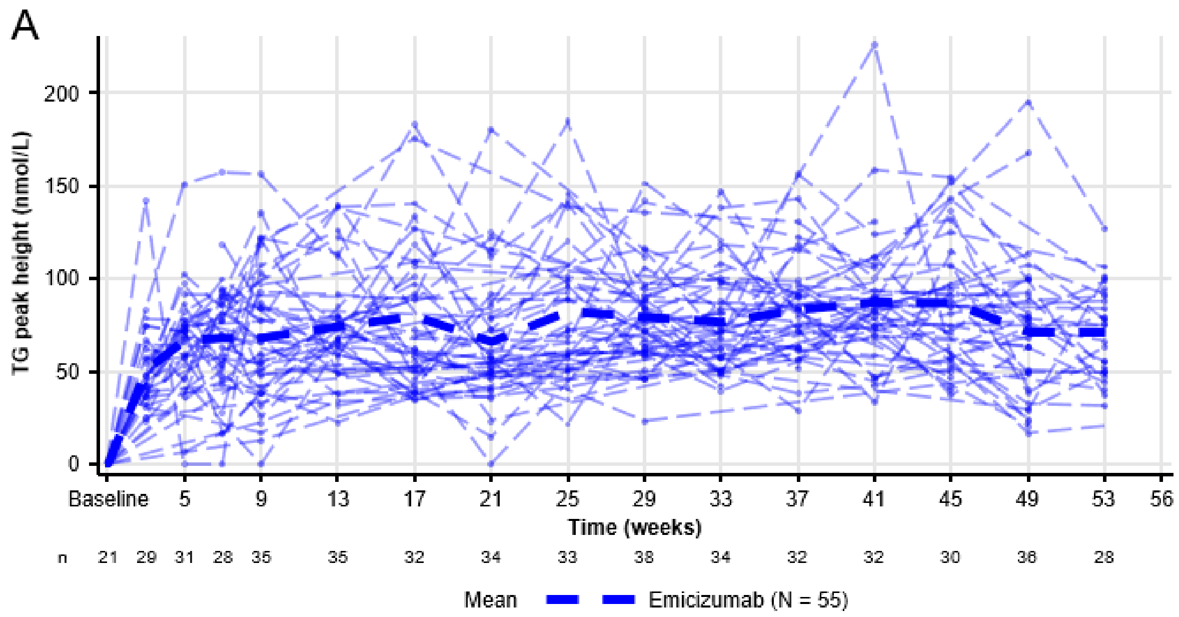

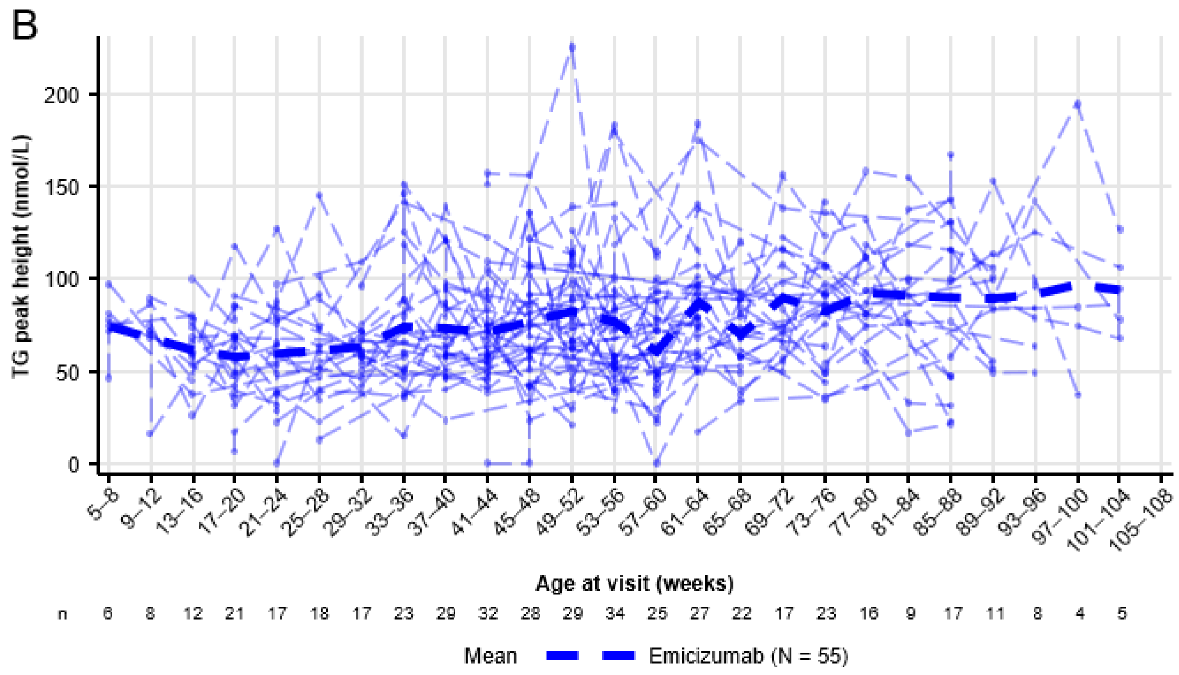

For the participant whose dose was up-titrated, only data before up-titration are included. For the analysis of TG peak height by age, only samples from week 5 onwards were considered. TG, thrombin generation.

Figure S6. Mean and individual FVIII-like activity (A) over time and (B) by age at visit during the maintenance period

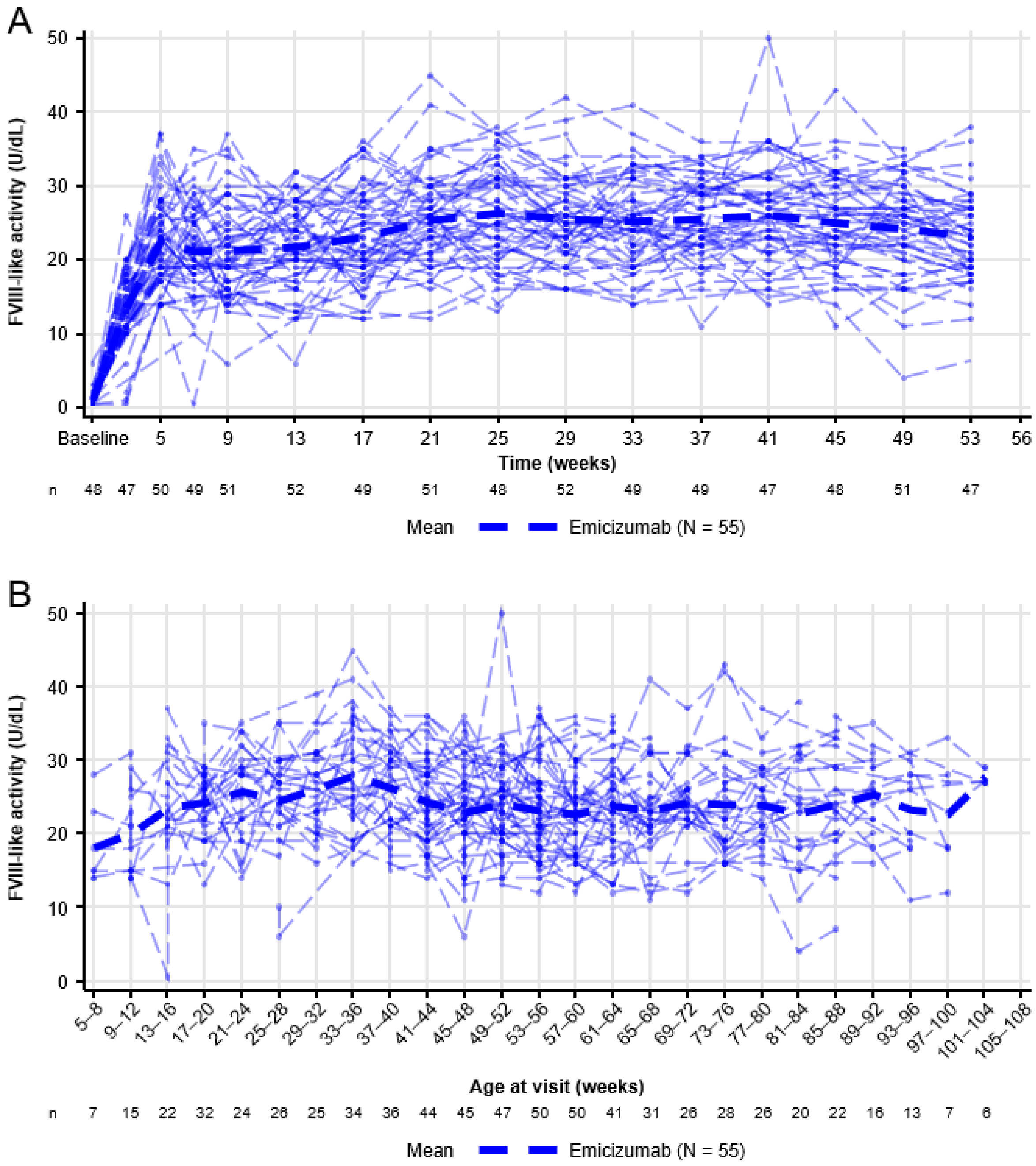

For the participant whose dose was up-titrated, only data before up-titration are included. For the analysis of FVIII-like activity by age, only samples from week 5 onwards (maintenance period) were considered. Values reported as <1 U/dL are replaced with 0.5 (LLOQ). FVIII, factor VIII; LLOQ, lower limit of quantification.
